# The Influence of Resin Infiltration on the Shear Bond Strength of Orthodontic Brackets: A Systematic Review and Meta-Analysis

**DOI:** 10.3390/jfb16010032

**Published:** 2025-01-17

**Authors:** Sylwia Kiryk, Jan Kiryk, Jacek Matys, Maciej Dobrzyński

**Affiliations:** 1 Department of Pediatric Dentistry and Preclinical Dentistry, Wroclaw Medical University, Krakowska 26, 50-425 Wroclaw, Poland; s.roguzinska@gmail.com (S.K.); maciej.dobrzynski@umw.edu.pl (M.D.); 2 Oral Surgery Department, Wroclaw Medical University, Krakowska 26, 50-425 Wroclaw, Poland; jan.kiryk@umw.edu.pl

**Keywords:** ICON, orthodontic brackets, resin infiltration, shear bond strength

## Abstract

The quality of the enamel plays a critical role in the retention and performance of orthodontic brackets. This systematic review and meta-analysis aimed to evaluate the effect of resin infiltration pretreatment on the shear bond strength (SBS) of orthodontic brackets. An electronic search was conducted in October 2024 using PubMed, Web of Science (WoS), and Scopus databases, employing the keywords (resin infiltration AND bracket); (ICON AND bracket). The review adhered to PRISMA guidelines and utilized the PICO framework. Of the 143 articles initially identified, 63 underwent screening. Strict inclusion criteria were applied of which the most important were resin infiltration pretreatment, studies conducted on natural teeth and SBS evaluation. This left 19 studies for final analysis. The risk of bias was assessed using the checklist for quasi-experimental studies (Non-Randomized Experimental Studies) developed by the Joanna Briggs Institute (JBI). Among these, 13 studies used human teeth and 13 utilized Transbond XT as the adhesive. Metal brackets were predominantly examined (*n* = 17). The Adhesive Remnant Index (ARI) was assessed in 13 studies. Importantly, 11 studies concluded that resin infiltration significantly enhances SBS, 8 of which were conducted on human teeth. The meta-analysis revealed significantly higher SBS results when resin infiltrate was applied to healthy enamel. This finding underscores the dual benefits of resin infiltration: increased bond strength and the protection of enamel integrity during debonding procedures. The results suggest that resin infiltration not only improves the mechanical retention of orthodontic brackets but also serves as an enamel-preserving approach.

## 1. Introduction

Enamel morphogenesis is a complex process that begins with the secretion of enamel matrix protein, followed by mineralization and maturation [1]. Abnormalities in these processes lead to enamel developmental disorders (DDE). These include hypomineralization, hypomaturation, hypoplasia, and hyperplasia. Their causes may be systemic, genetic, or environmental, such as fluorosis, amelogenesis imperfecta, or vitamin D deficiency. Hypomineralization and hypomaturation are qualitative defects and occur due to disorders in the maturation phase of amelogenesis. [2]. Hypomineralization manifests itself as a soft, chalky, or cheese-like appearance of the enamel, while hypomaturation presents as opaque and discolored enamel that fractures easily [3]. There is usually a clear border with healthy tissue [4]. Hypoplasia is a quantitative defect caused by abnormal thickness of the enamel [5]. It manifests itself as pitting, fissuring, or absence of enamel. In most of these cases of DDE the bond with the adhesive material is disturbed. That makes tooth reconstruction and bonding of orthodontic brackets difficult. This is believed to be due to the shielding of the enamel mineral by acid-insoluble proteins [6]. Hyperplasia, on the other hand, is characterized by the presence of enamel rings protruding beyond the normal surface of the crown on all erupted teeth [7]. The incorrect shape of the vestibular surface of the teeth makes it difficult not only to bond, but also to position the orthodontic brackets correctly.

**Figure 1:**
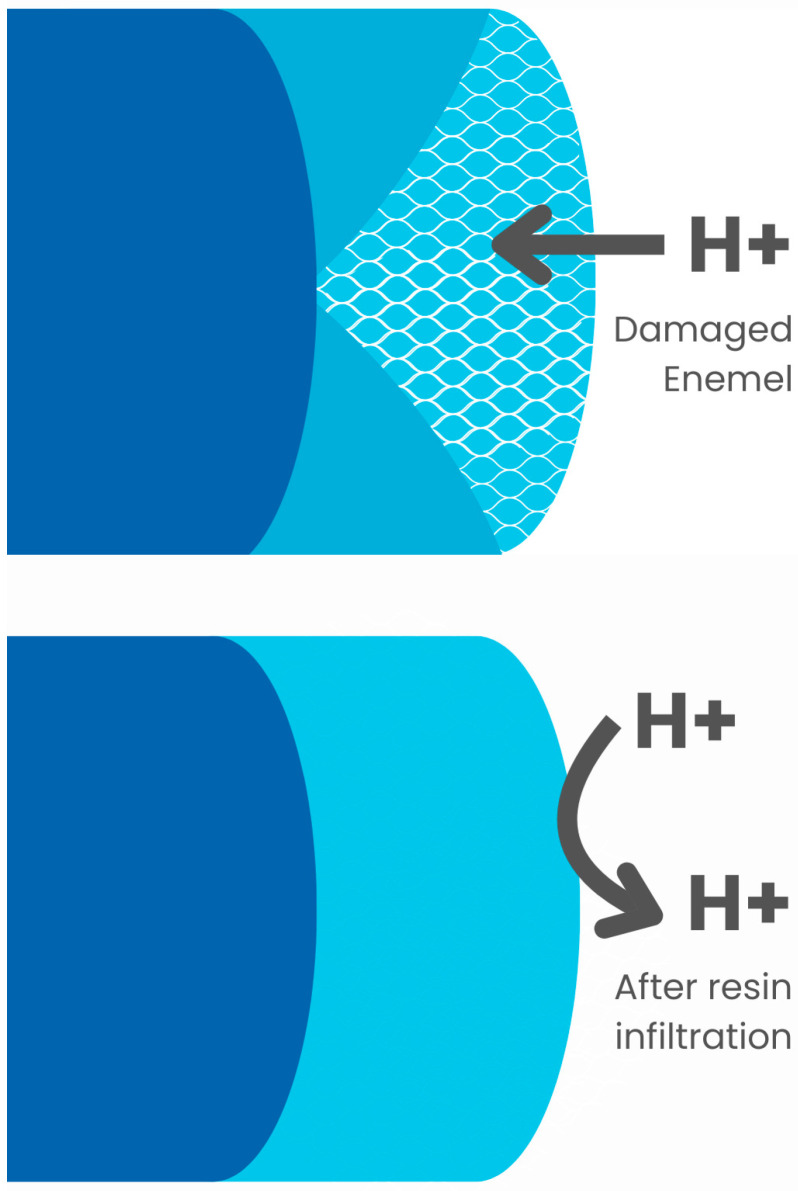
Resin infiltration.

The need for treatment, preventing further damage and improving aesthetics, has led to the development of several noninvasive methods for treating noncavity carious lesions and DDE, including fluoride, casein phosphopeptide, and amorphous calcium phosphate [8]. A relatively new product that combines prevention with restoration is the resin infiltration agent [9]. It penetrates demineralized enamel lesions and closes intercrystalline spaces by creating a polymer skeleton. That micromechanically blocks remaining enamel prisms and hydrogen ions, preventing further demineralization and caries development while increasing microhardness [9,10,11]. Due to the low viscosity of the resin, the pores between the crystals are filled, creating a diffusion barrier within the entire lesion (Figure 1). It leads to hardening of the demineralized tissues and increasing their mechanical strength [10]. This type of treatment is often used in young patients who often need orthodontic care. Therefore, the question of how it will affect subsequent treatment with fixed orthodontic appliances, which is a challenge in patients with demineralization and DDE due to adhesion problems, is very important.

Accidental debonding of an orthodontic bracket during treatment with fixed appliances can lead to longer treatment times, higher treatment costs, frustration, and dissatisfaction for both the patient and the dentist, increase the risk of white spots appearing and, in some cases, even to uncontrolled tooth movement [12,13,14,15,16]. That is why it is so important that the brackets stay on the teeth throughout the entire treatment process. There are numerous scientific studies analyzing the factors that affect the detachment of brackets. Some authors divide them into factors dependent and independent of the patient [17,18,19]. Others, distinguish early and late factors [20]. As observations show, most accidental detachments of brackets occur immediately after their gluing. This is related to errors in the procedure of gluing the brackets and the duration of full polymerization of the composite, which can last from 30 min to 24 h [21,22,23,24]. Late failures are caused by mechanical damage to the device by the patient. They most often result from non-compliance with recommendations, especially dietary ones, aging of the composite material, and the formation of white spots around the orthodontic brackets, which weaken the bond of the adhesive material with the enamel [20,25]. Unfortunately, in some cases, orthodontic treatment must be continued or even started despite the presence of enamel demineralization.

**Figure 2:**
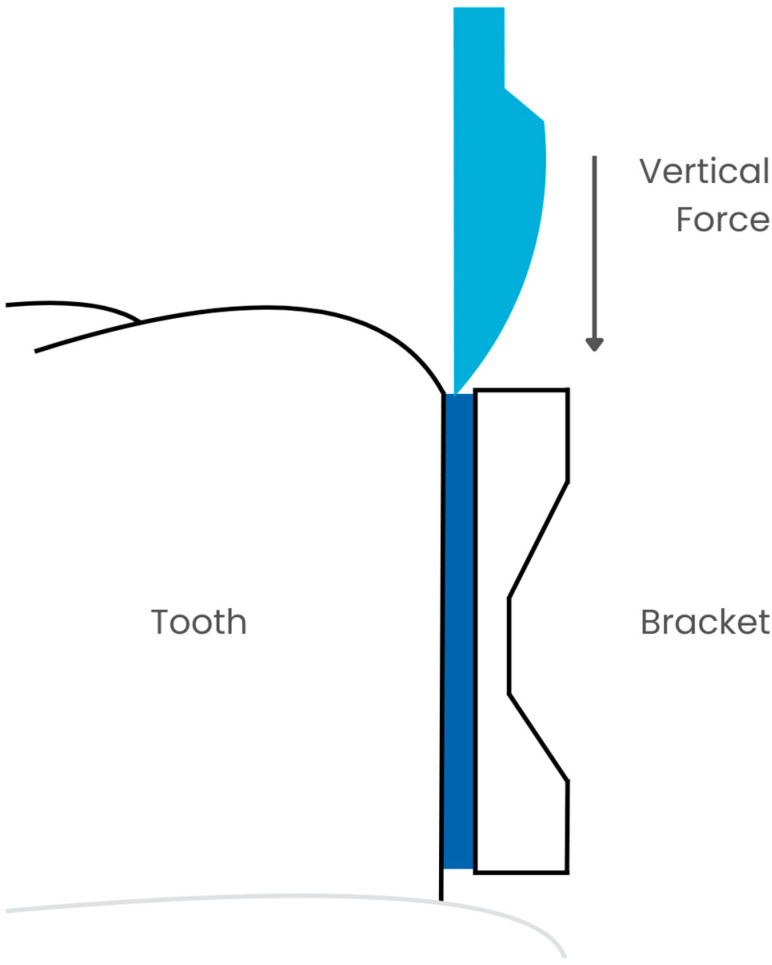
SBS measurement.

Despite the additional difficulty in maintaining the brackets on the teeth due to the reduced SBS to demineralized enamel, there is also an increased risk of enamel damage during debonding of the brackets [26]. It should be remembered that after the orthodontic treatment is completed, the brackets must be removed without damaging the enamel. Therefore, during bonding, the goal should not be to achieve maximum bond strength, but to proceed with great caution. According to research, the minimum shear bond strength (SBS) that allows the bracket to be held throughout the treatment process should be between 6 and 8 MPa [27]. Other authors report that even at a force of 10 MPa, there is a risk of damaging the enamel during debonding [28,29] (Figure 2).

Due to the constant increase in demand for orthodontic treatment, patients with DDE are increasingly appearing in offices. It is extremely important to be aware of how to provide them with the most comfortable treatment while minimizing the risk of enamel damage during removal of the appliance, which is much greater than in healthy patients. However, the authors of this systematic review did not identify any previous reviews assessing the impact of Resin Infiltrant Agents on the shear bond strength of orthodontic brackets. This study aims to address this gap.

## 2. Materials and Methods

### 2.1. Focused Question

The systematic review followed the PICO framework [30] as follows: In the case of orthodontic bracket bonding (population), will the use of resin infiltration (investigated condition) affect shear bond strength (outcome) compared to bonding without enamel pretreatment (comparison condition)?

### 2.2. Protocol

**Figure 3:**
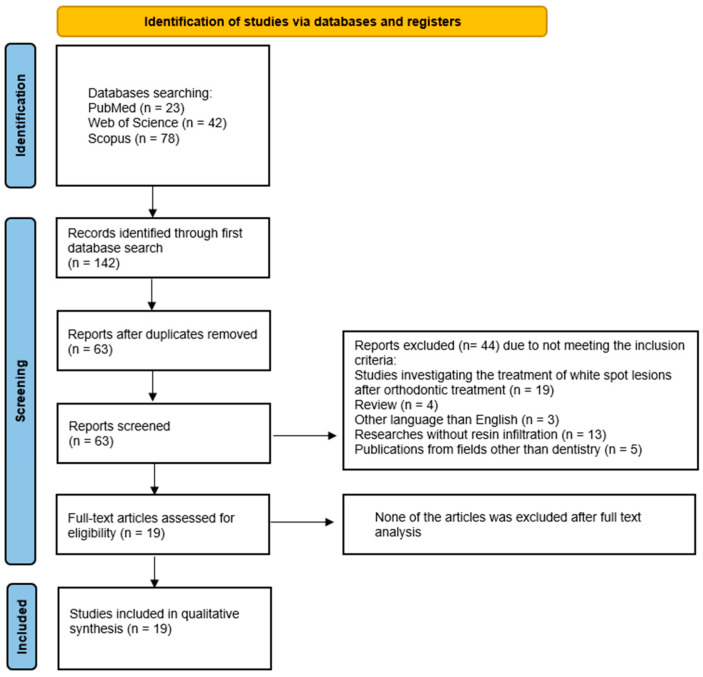
The PRISMA 2020 flow diagram.

The selection process for articles included in the systematic review was carefully outlined following the PRISMA flow diagram [31] (see Figure 3). The systematic review was registered on the Open Science Framework under the following link: https://doi.org/10.17605/OSF.IO/CRK2W (accessed 5 October 2024).

### 2.3. Eligibility Criteria

The researchers agreed to include only the articles that met the following criteria [32,33,34,35,36,37,38,39,40]:

- Resin infiltration pretreatment;
- Studies conducted on natural teeth;
- SBS evaluation studies;
- In vitro and in vivo studies;
- Use all kinds of orthodontic brackets;
- Studies in English;
- Full-text articles.

The exclusion criteria the reviewers agreed upon were as follows [32,33,34,35,36,37,38,39,40]:

- No resin infiltration pretreatment;
- No SBS evaluation;
- Non-English papers
- Systematic review articles;
- Review articles;
- No full-text accessible;
- Duplicated publications.

No restrictions were applied with regard to the year of publication.

### 2.4. Information Sources, Search Strategy, and Study Selection

In October 2024, the PubMed, Scopus, and Web of Science (WoS) databases were searched to find articles meeting the specified inclusion criteria. To find articles focusing on the influence of resin infiltration pretreatment on shear bond strength of orthodontic brackets, the search was narrowed to specific keywords: (resin infiltration AND bracket); (ICON AND bracket). In the Scopus and WoS database, the results were refined to titles, abstracts, and keywords, while in PubMed, they were narrowed down to titles and abstracts. All searches conformed to the predefined eligibility criteria, and only articles with accessible full-text versions were taken into consideration.

### 2.5. Data Collection and Data Items

Two reviewers (J.K. and S.K.) carefully selected the articles that met the inclusion criteria. The extracted data were then introduced into a standardized Excel file.

### 2.6. Assessing Risk of Bias in Individual Studies

In the preliminary phase of selecting studies, the authors independently examined the titles and abstracts of each study to reduce the possibility of reviewer bias. They evaluated the level of consensus among reviewers using Cohen’s κ test [41]. The authors resolved any disagreements about whether to include or exclude a study through discussions.

### 2.7. Quality Assessment

Two independent reviewers (J.M. and M.D.) assessed the procedural quality of each study included in the article using the Joanna Briggs Institute (JBI) checklist for quasi-experimental studies (nonrandomized experimental studies) [42]. The checklist consists of 9 questions:

- Is it clear in the study what is the ‘cause’ and what is the ‘effect’?
- Were the participants included in any similar comparisons?
- Were the participants included in any comparisons receiving similar treatment/care, other than the exposure or intervention of interest? Was there a control group?
- Were there multiple measurements of the outcome both before and after the intervention/exposure?
- Was a follow up completed, and if not, were differences between groups in terms of their follow up adequately described and analyzed? Were the outcomes of participants included in any comparisons measured in the same way?
- Were the outcomes measured in a reliable way?
- Was an appropriate statistical analysis used?

The answer to these questions can be yes, no, unclear, not applicable. Any discrepancies in answering were resolved through discussion until a consensus was reached.

### 2.8. Meta-Analysis

Sheer bond strength was calculated in some of the reviewed studies and could be compared with the meta-analytic tools. The publications were divided depending on whether the resin infiltrates healthy or demineralized enamel. In order to compare the raw mean differences between the treated and control groups, the researchers applied forest plots. In order to detect potential publication bias, the researchers evaluated funnel plots, along with rank correlation tests and regression tests.

The degree of heterogeneity (tau^2^) was estimated using restricted maximum likelihood [43]. In addition to the tau^2^ estimate, the Q-test for heterogeneity and the I^2^ statistic were also used. Analyses were performed separately for studies made on sound and demineralized enamel.

Statistical analysis was performed with Jamovi 2.3.28 [44], supported by the R statistical environment [45] with the MAJOR package [43].

In order to compare the raw SBS values between studies conducted on demineralized and sound teeth, the weighted mean was compared using Welch’s *t*-test.

## 3. Results

### 3.1. Study Selection

A search of the electronic databases PubMed, Scopus, and WoS yielded 143 records. Of these, 80 were duplicates and were thus removed. The remaining 63 articles were subjected to abstract screening, which resulted in the exclusion of 44 articles that did not meet the inclusion criteria: 19 studies were investigating the treatment of white spot lesions after orthodontic treatment, 4 studies were review, 3 studies were written in other languages than English, in 13 studies the resin infiltrant was not in use, and 5 studies were from other fields than dentistry. A thorough analysis of the 19 confirmed that all of them met the inclusion criteria. Therefore, the final number of articles included in this review was 19.

### 3.2. General Characteristics of the Included Studies

The studies included in the systematic review present research focused on the shear bond strength of orthodontic brackets bonded to the enamel pretreated with resin infiltrants. The general characteristics of the studies are presented in Table 1. Thirteen researchers decided to conduct the experiment on human teeth [46,47,48,49,50,51,52,53,54,55,56,57,58], while the rest used bovine teeth [59,60,61,62,63,64]. In 13 studies, Transbond XT was used as the only adhesive material [46,48,52,53,54,55,56,57,58,60,61,63,64]. Four authors conducted tests on several adhesive materials and compared them with each other [47,49,50,59], and two did not provide the name of the agent used [51,62]. Seventeen authors examined metal brackets [46,47,48,49,50,51,52,53,55,57,58,59,60,61,62,63,64], while the remaining two did not provide information about the material of the brackets used [54,56].

The largest number of authors, 10, decided to compare SBS after covering the enamel with various preparations inhibiting demineralization [46,48,52,53,54,56,57,60,61,62]. Three of them conducted studies on bovine teeth [60,61,62]. Triwardhani et al. observed the highest SBS values on resin-infiltrated teeth, but at the same time he received the highest ARI value [62]. Despite this, he recommends infiltration. On the other hand, the results of Attin et al. [60] and Vianna et al. [61] suggest that infiltration does not affect SBS. From a clinical point of view, more important are studies conducted on human teeth. Among them, only Gulec et al. concluded that resin infiltration reduces the SBS value, but they additionally checked the content of mineral substances in the tooth tissues and after infiltration it was increased [46]. Costenoble et al. decided to bond the brackets immediately after infiltration and a month later [56]. Their results show that in the case of waiting a month, the SBS value becomes significantly lower, which does not happen when the procedures are performed immediately after each other. The remaining authors agree that infiltration increases the SBS, and its value is similar to that obtained on healthy enamel or even higher [48,52,53,54,57]. Ekizer et al. [54] and Baka et al. [48] showed that a similar effect can be obtained using CPP-ACP, which was not confirmed by Nimbalkar et al. [57].

Naidu et al. [59] and Mews et al. [63] used resin infiltration not only on demineralized enamel but also on healthy enamel and came to the same conclusions that even on healthy enamel there is an increase in bond strength and, in addition, despite the increase in ARI, there are fewer enamel defects after debonding.

Montasser and Taha [55] and Al-Mayali [50] studied the effect of different bonding agents on SBS after resin infiltration. Their results are completely different. While Montasser and Taha [55] obtained the lowest SBS values with ICON and self-etching primer, Al-Mayali [50] obtained the highest bond strength with this combination. However, both studies have one thing in common—resin infiltration causes more adhesive to remain on the enamel. Yetkiner et al., in addition to using different bonds, also checked the effect of H_3_PO_4_ and HCl etching, but found no significant differences with SBS [64].

Insee et al. [47] and Anicic et al. [49] investigated whether the adhesive used to bond brackets affects SBS when infiltration is used. It turned out to be not insignificant. However, their results were contradictory. Insee et al. [47] obtained a decrease in SBS with Assure Plus, while Anicic et al. [49] obtained the highest bond strength with this adhesive. However, both obtained results similar to the control group with Transbond XT.

Interesting and original studies were conducted by Al-Mayali et al. [51] and Hammad et al. [58]. They investigated how diet affects the SBS of brackets after resin infiltration. Al-Mayali et al. [51] used the storage of teeth in ethanol and corn oil and concluded that despite the significant negative impact of these substances, especially alcohol, on SBS, infiltration causes an increase in bond strength regardless of the conditions. Hammad et al. [58], examining in vivo the effect of Coca-Cola and Sprite beverages on teeth intended for extraction, reached identical conclusions.

### 3.3. Main Study Outcomes

The detailed characterization of selected studies is presented in Table 2. Publications varied with each other in terms of the assessed parameters. Thirteen authors evaluated the composite remaining on the teeth using ARI [46,47,48,49,50,53,54,55,56,59,62,63,64], and for the purpose of this review, the arithmetic mean was calculated to compare the results. It turned out that in three works it was over 1 [46,53,54], and in another three over 2 [47,55,63]. SEM analysis was performed for six studies [48,52,56,58,62,64], and only in one of them damage to the enamel surface was observed, and this study used bovine teeth [62]. Only in three works was the SBS value below 10 MPa [46,47,62]. Eleven researchers concluded that resin infiltration significantly increases SBS value [48,49,50,51,52,54,57,58,59,62,63]; eight of these studies were conducted on human teeth [48,49,50,51,52,54,57,58].

### 3.4. Quality Assessment

For all of the 9 questions, 14 papers received a positive answer to 8 of them [46,47,48,49,50,51,52,53,54,55,56,57,60,61], 4 papers received a positive answer to 7 [58,62,63,64], and 1 remaining paper received a positive answer to 6 of them [59] (see Table 3).

### 3.5. Meta-Analysis

The raw data used for the meta-analysis, along with the division into groups, are presented in Table 4.

**Figure 4:**
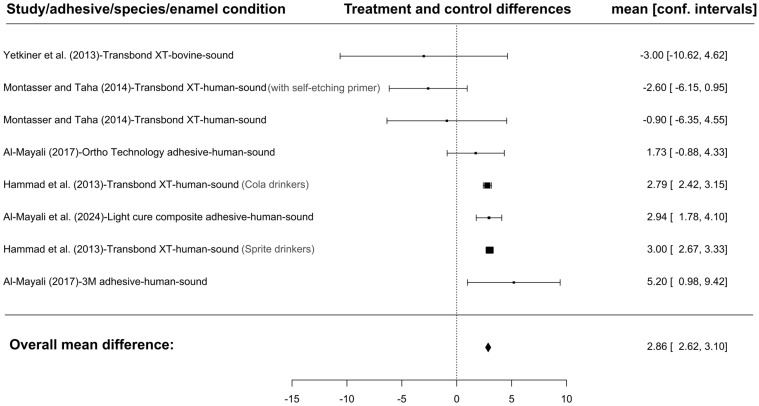
The forest plot for SBS is shown below. A total of eight groups were assessed because five of the studies included multiple groups relevant to the current analysis, which were presented separately. For each study, the absolute differences between the means for the treated and untreated groups (represented by the black rectangles), and their confidence intervals are displayed. The size of the rectangle corresponds to the number of evaluated teeth. The dashed line in the middle represents the ‘point of no effect’. The results on the left side of the plot show studies in which the treated group had a lower SBS value compared to the control group, while those on the right side indicate higher values [50,51,55,58,64]. The figure was created with Jamovi 2.3.28 (Jamovi, Australia) software.

Meta-analysis showed that when resin infiltration of healthy enamel was used, SBS values were significantly higher in the control groups. Eight studies conducted on sound enamel were included in the analysis (Figure 4). Mean differences ranged from −3.0000 to 5.2000, with 62% of the estimates being positive. The estimated mean difference based on the random effects model was \hat{\mu} = 2.8643 (95% CI: 2.6249 to 3.1037). The mean score was significantly different from zero (z = 23.4512, *p* < 0.0001). According to the Q test, the true scores appeared to be heterogeneous (Q(7) = 15.9369, *p* = 0.0257, tau^2^ = 0.0000, I^2^ = 0.0006%). The 95% prediction interval for the true scores is given by 2.6249 to 3.1037. Hence, the true results of the studies are in the same direction as the estimated mean result, even though some heterogeneity may exist. Two groups (Hammad et al. (2013)-Transbond XT-human-sound.1; Hammad et al. (2013)-Transbond XT-human-sound.2 [58]) had relatively large weights compared to the rest of the studies (i.e., \mbox{weight} \ge 3/k, so a weight at least 3 times as large as having equal weights across studies). An examination of the studentized residuals revealed that one study (Montasser and Taha (2014)-Transbond XT-human-sound.2 [55]) had a value larger than ±2.7344 and may be a potential outlier in the context of this model. According to the Cook’s distances, none of the studies could be considered to be overly influential.

**Figure 5:**
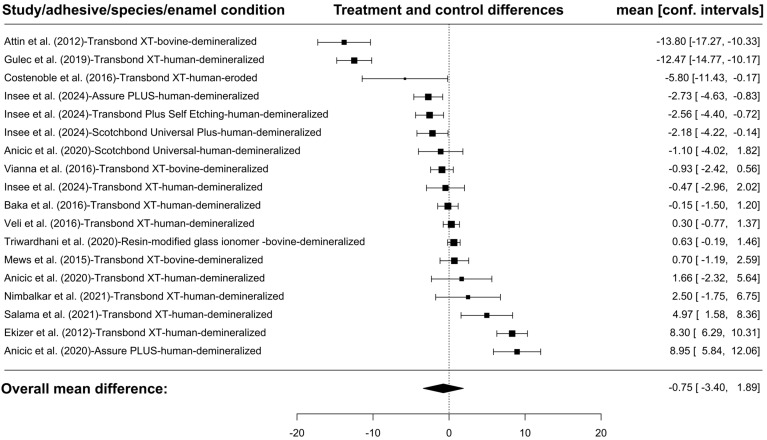
The forest plot on SBS is shown below. The final number of groups assessed was 18 because some of the 13 studies assessed included multiple groups relevant to the current analysis and were presented separately. For each study, the absolute differences between the mean for the treated and untreated groups (black rectangle) and the confidence intervals are shown. The size of the rectangle refers to the number of evaluated teeth. The dashed line in the middle shows the ‘point of no effect’. The results on the left-hand side of the table illustrate studies in which the treated group exhibited a lower SBS value in comparison to the control group, while those on the right-hand side demonstrate higher values [46,47,48,49,52,53,54,56,57,60,61,62,63]. The figure was created with Jamovi 2.3.28 (Jamovi, Australia) software.

The meta-analysis showed that in the case of resin infiltration of demineralized enamel there were no significant differences in the SBS level between the study and control groups. A total of 18 groups presenting results conducted on demineralized enamel were included in the analysis (Figure 5). The observed mean differences ranged from −13.8000 to 8.9500, with the majority of estimates being negative (56%). The estimated average mean difference, based on the random-effects model, was μ^ = −0.7533\hat{\mu} = −0.7533 (95% CI: −3.3999 to 1.8933). Therefore, the average outcome did not differ significantly from zero (z = −0.5579, *p* = 0.5769). According to the Q-test, the true outcomes appeared to be heterogeneous (Q(17) = 308.9387, *p* < 0.0001, tau^2^ = 30.8538, I^2^ = 97.1450%). A 95% prediction interval for the true outcomes ranged from −11.9573 to 10.4506. Hence, although the average outcome is estimated to be negative, in some studies the true outcome may, in fact, be positive. No study had a value greater than ±2.9913 in the examination of the studentized residuals, indicating the absence of outliers in the context of this model. No study was considered overly influential according to Cook’s distances. Neither the regression test nor the rank correlation showed any asymmetry in the funnel plot (*p* = 0.6540 and *p* = 0.7099, respectively).

**Figure 6:**
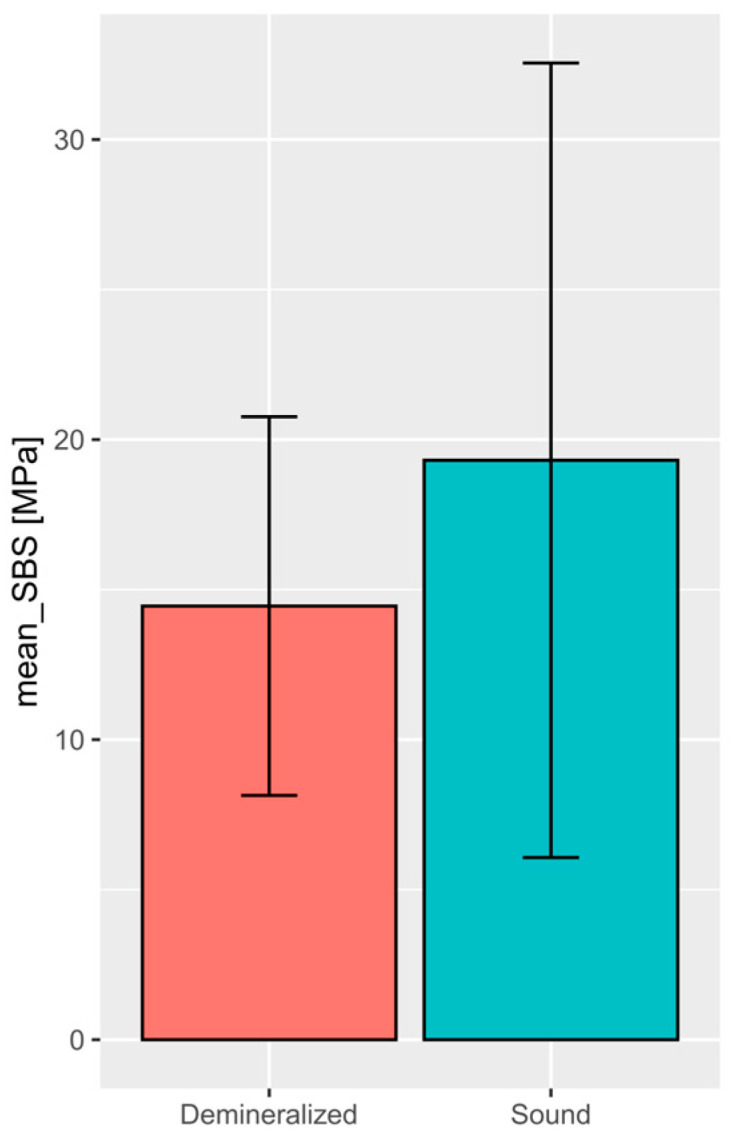
The following plot provides a comparison of the raw values of SBS among studies on demineralized and sound teeth. The plot shows weighted means and standard deviations, which reflect the different size of populations included in the studies. The difference presented is statistically significant (Welch’s *t*-test *p*-value = 0.0002) [65]. The figure was created with Jamovi 2.3.28 (Jamovi, Australia) software.

A comparison of SBS values between healthy and demineralized enamel showed that significantly higher values occurred in the case of healthy enamel. (See Figure 6).

## 4. Discussion

The aim of this systematic review was to determine the effect of resin infiltration on the bond strength of orthodontic brackets. Eleven researchers found that resin infiltration significantly increased the SBS value [48,49,50,51,52,54,57,58,59,62,63]; eight of these studies were conducted on human teeth [48,49,50,51,52,54,57,58]. The analysis showed that the use of infiltration has no significant effect on the SBS of demineralized enamel; what is more, in the control groups the SBS was higher than after pretreatment of healthy enamel. Thirteen authors evaluated the composite remaining on the teeth using ARI [46,47,48,49,50,53,54,55,56,59,62,63,64]. Of these, six obtained a medium result below 1 [48,49,50,56,59,64]. No correlation was found between SBS and ARI values. SEM analysis was performed for six studies [48,52,56,58,62,64], and only in one of them damage to the enamel surface was observed. Additionally, it was a study using bovine teeth [62].

A few authors in their publication stated that at SBS above 10 MPa during debonding of orthodontic brackets, damage to the enamel may occur due to detachment of its prisms [28,29]. It is therefore alarming that after infiltration only three authors obtained SBS below 10 MPa [46,47,62]. In seven studies this result was within the range of 10–15 MPa [47,48,49,50,51,58,61], and in the remaining ones it was even higher [49,50,52,53,54,55,56,57,63,64]. Articles in which different adhesive materials were used show that the material used has a large effect on SBS. Al-Mayali, who studied adhesives from Ortho Technology and 3M, obtained a difference in bond strength of 7.78 MPa [50]. Insee et al. obtained the highest SBS values for Transbond XT material [47], similarly in the case of the study conducted by Demirsoy et al. without the use of infiltration, they obtained an average SBS value for Transbond XT of 15.03 MPa [66]. In contrast, in the study of Anicic et al. the bond strength for Transbond XT was 7.29 MPa lower than for Assure Plus [49]. This only proves the need for further research. However, according to the results of the meta-analysis, even after resin infiltration, the SBS value of demineralized enamel is significantly lower than that of healthy enamel.

Four researchers conducting experiments on bovine teeth determined the ARI [59,62,63,64]. Their mean results ranged from 0 [64] to 2.4 [63], which is significantly better than the values obtained in studies without the use of ICON, where the mean obtained by da Rocha et al. was 2.73 [67], while Henkin et al., using metal brackets from different companies, obtained mean results from 0.47 to 2.4, depending on the brackets used [68]. In the case of human teeth, ARI was measured in nine studies [46,47,48,49,50,53,54,55,56]. The lowest mean score of 0.5 was obtained by Costenoble et al. and Baka et al. [48,56], while the highest was 2.7 by Montasser and Taha [55]. In studies without resin infiltration, the results were lower. Mean values from 0.92 to 1.67, depending on the brackets used, were declared by Cervantes-Ganoza et al. [69], and Nawrocka et al. obtained mean scores from 1.9 to 2 [70]. Lower ARI scores are more beneficial in orthodontics because the need to remove remaining adhesive is an additional risk factor for damage to the enamel surface [71].

Assessment of the enamel surface using Scanning Electron Microscope (SEM) after the debonding of brackets allows for the determination of whether one of the important goals of research on shear bond strength has been achieved, namely minimizing enamel damage [72]. According to the research of Lishna et al., after debonding of metal brackets, in 25% of cases a rough surface, numerous thick scratches, and fine grooves are visible in SEM [73]. Among the works qualified for review, the SEM assessment was only descriptive without providing the Enamel Demage Index (EDI), which makes a reliable assessment difficult. Triwardhani et al. noticed damage in the form of irregular enamel porosity and damaged honeycombs, but there were fewer of them than in the control group [62]. However, Yetkiner et al. and Hammad et al. did not notice any enamel damage in the SEM image [58,64].

The need for research on ceramic brackets is immediately apparent, especially since Bakhadher in his review indicated ceramic brackets as a factor for increasing shear bond strength, and at the same time increasing the risk of enamel damage during debonding [74]. It may also be important to examine SBS during debonding using a different force direction. All of the researchers tested the force parallel to the enamel surface, and the studies by Klocke and Kahl-Nieke show that the force direction significantly affects SBS [75]. It would be worthwhile to examine the enamel surface after debonding using SEM with EDI to enable a reliable comparison of results. Additionally, it cannot be ignored that individual researchers who decided to conduct tests on various adhesive materials proved that SBS is dependent on them. However, there are too few studies to draw reliable conclusions.

Our study has several limitations that should be acknowledged. A meta-analysis for Adhesive Remnant Index (ARI) could not be conducted due to its ordinal nature and the lack of uniformity in the scales used across studies, with some employing a 0–3 scale and others a 1–4 scale. Although we standardized these scales to enable comparisons by presenting results as percentages, this approach may not fully capture the nuances of ordinal data or allow for advanced statistical analyses. Additionally, the interpretation of Scanning Electron Microscope (SEM) results posed a challenge, as only a limited number of studies provided SEM data, and several relied on the reader’s interpretation of the images without detailed descriptions. These inconsistencies limited the depth of analysis and comparability of the results. Furthermore, the division of studies into those conducted on healthy and demineralized enamel, while necessary for meaningful comparisons, introduced methodological heterogeneity. Lastly, the reliance on percentage representation for ARI and the variability in study designs underscore the need for more standardized methodologies in future research to enhance data comparability and the robustness of meta-analyses.

## 5. Conclusions

The presented studies allow us to clearly state that the use of ICON has a positive effect on orthodontic treatment by increasing shear bond strength while protecting the enamel from damage during debonding. There is no correlation between SBS and ARI values. The obtained results indicate that the use of resin infiltration of enamel before orthodontic treatment with fixed appliances can have significant clinical benefits. First of all, it reduces the risk of enamel damage during debonding in patients with DDE. Additionally, it can be used in patients who have difficulties in following dietary recommendations and, therefore, often experience accidental debonding of brackets, which often results in a significant extension of treatment time.

## Figures and Tables

**Table 1:** General characteristic of the included studies.

Study	Aim of the Study	Materials and Methods	Results	Conclusions
Al-Mayali [50]	To investigate the difference in SBS of orthodontic brackets after the use of caries-infiltrating resin and its dependence on the type of bonding used.	Forty-eight teeth were divided into four groups, and the force required to remove the brackets was recorded. The remaining adhesive material was examined using light microscopy.	The highest bond strength was achieved using infiltration with self-etching bond. More adhesive remained on the infiltrated enamel surfaces.	The use of the ICON system increases the bond strength between the tooth and the orthodontic bracket.
Al-Mayali et al. [51]	To investigate the effect of food simulants on the bond strength of orthodontic brackets after the use of ICON.	Braces were bonded to 48 teeth, half of which were coated with ICON. They were then divided into three groups and stored in distilled water, 50% ethanol, and corn oil for 30 days, respectively.	Teeth coated with ICON had higher SBS regardless of environment.	The use of ICON increases the bond strength, while food, especially ethanol, reduces it.
Anicic et al. [49]	Evaluation of the bond strength of orthodontic brackets bonded with different adhesives to demineralized enamel treated with low-viscosity infiltration resin.	Thirty-six teeth were demineralized and divided into three groups: Group II: ICON + Transbond XT; Group III: ICON + Scotchbond Universal; Group IV: ICON + Assure PLUS. Group I—control with sound enamel.	The highest SBS was shown by the ICON + Assure PLUS group.	The use of infiltrating resin does not worsen SBS compared to healthy enamel; it may even increase it in combination with Assure PLUS.
Attin et al. [60]	Comparison of the effect of demineralized and differently treated demineralized enamel on the SBS of orthodontic brackets.	Sixty enamel samples were assigned to five groups. G I: control. The rest were demineralized. In G III Elmex Gelee, 1.23% F was used; G IV: Clinpro White Varnish, 2.23% F G V: ICON.	The highest SBS was obtained in the control group, followed by the group using ICON.	Infiltration is beneficial when it is necessary to use orthodontic brackets on demineralized enamel.
Baka et al. [48]	To compare the effects of different remineralization procedures on tooth surface roughness, SBS, and ARI of a self-etching primer used for bonding orthodontic brackets.	A total of 140 teeth were divided into 7 groups. One remained as the control, and the rest of the teeth were demineralized and treated with CPP-ACP, fluoride, a microabrasion mixture, a microabrasion agent, and resin infiltration.	The control, CPP-ACP and resin infiltration groups did not show any statistically significant differences in SBS values.	Remineralization treatments restore the reduced SBS value of orthodontic brackets and reduce the surface roughness caused by enamel demineralization.
Costenoble et al. [56]	Investigation of SBS of orthodontic brackets bonded to damaged enamel treated prophylactically and analysis of enamel-bracket connections.	Ninety-one brackets were bonded to enamel divided into seven groups: healthy, eroded (E); E + calcium silicate and sodium phosphate (CSP); E + ICON; E + ICON after one month; E + experimental resin; and E + experimental resin after one month.	CSP and infiltration samples had the same SBS as the control group, but delayed bracket placement had lower SBS values. ARI scores were lowest after infiltration.	The use of CSP or infiltration prior to bonding the brackets does not negatively affect the bond strength, but it should be performed shortly after resin infiltration.
Ekizer et al. [54]	To investigate and compare the effect of different demineralization inhibition methods on SBS and fracture site of orthodontic adhesive.	Eighty teeth were divided into four groups and demineralized. Group 2 received ICON, group 3 APF, and group 4 CPP-ACP/wF. Then, the brackets were bonded.	Significantly higher SBS values were observed in the resin-infiltrated group and the CPP-ACP/wF-treated group.	The action of the resin and the application of CPP-ACP/wF increases the SBS compared to untreated, demineralized enamel.
Gulec et al. [46]	To investigate the effectiveness of CPP-ACP gel and resin infiltration on decalcified enamel and to explain the correlation, if any, between ion release capacity and SBS.	Eighty teeth were divided into four groups: Group I control, Group II demineralization only, Group 3 demineralization + CPP-ACP gel and Group 4 demineralization + resin infiltrate.	Both infiltration and CPP-ACP gel resulted in a decrease in SBS values. There was no correlation between SBS and changes in the Ca/P ratio.	CPP-ACP and resin infiltration therapies increase tooth mineral content but produce lower SBS values.
Hammad et al. [58]	In vivo evaluation of the effect of two acidic carbonated beverages on the SBS of metal orthodontic brackets with and without resin infiltration.	Thirty patients scheduled for premolar extraction were divided into two groups based on the carbonated drink tested. ICON was used in each group before bonding the brackets.	The Coca-Cola group, which did not use ICON, showed the lowest SBS value.	Resin infiltration shows significant improvement in SBS, regardless of the type of beverage consumed.
Insee et al. [47]	To evaluate the SBS of orthodontic enamel brackets following initial resin infiltration treatment using different adhesive systems.	The sixty teeth were divided into five groups. Group I: healthy enamel + Transbond XT; group II: ICON + Transbond XT; group III: ICON + Scotchbond Universal Plus; group IV: ICON + Assure PLUS; group V: ICON + Transbond Plus Self Etching.	The SBS of the ICON + Assure PLUS and ICON + Transbond Plus Self Etching groups was significantly lower than that of the control and ICON + Transbond XT groups.	The SBS value after enamel infiltration depends on the adhesive system used.
Mews et al. [63]	To investigate the differences in SBS of orthodontic brackets on enamel surfaces of different mineralization after the use of an infiltrating agent or a conventional bonding agent.	A total of 320 bovine teeth were assigned to 8 groups, and the shear force required for debonding was recorded. Adhesive residue was assessed by light microscopy using ARI.	Resin-infiltrated surfaces showed the highest SBS and ARI values, and the fewest enamel defects.	Enamel infiltration applied before the placement of brackets has a protective effect, especially on demineralized enamel.
Montasser and Taha [55]	To investigate the effect of two enamel protective agents on the SBS of orthodontic brackets bonded using conventional and self-etching adhesive systems.	ICON or Clinpro was used on the teeth and the brackets were bonded using Transbond Plus Self Etching Primer + Transbond XT adhesive or 37% phosphoric acid etch + Transbond XT primer + Transbond XT adhesive	The lowest SBS value was obtained using ICON and the self-etching adhesive system.	The type of adhesive system affects SBS, while enamel infiltration affects the adhesive residue after bracket removal.
Naidu et al. [59]	To investigate the effect of enamel infiltration on SBS of resin cements for orthodontic teeth on healthy and demineralized enamel.	The brackets were bonded to healthy or artificially demineralized bovine enamel samples using different orthodontic adhesives and preceded by enamel infiltration in half of the cases.	ICON system increased the SBS of Transbond XT, Transbond Plus, and Fuji Ortho in healthy samples, and all except the Concise system on eroded enamel.	Infiltration of both sound and demineralized enamel increases the SBS of most orthodontic resin cements.
Nimbalkar et al. [57]	Comparison of the effect of fluoride varnish, CPP-ACPF and infiltrating resin on SBS of adhesive materials used to bond orthodontic brackets to demineralized enamel.	Sixty teeth were demineralized and divided into groups: Group I: control; group II: Duraphat; group III: CPP-ACPF; group IV: ICON.	The highest SBS values were obtained on resin-infiltrated enamel.	It is beneficial to use ICON as a preventive measure before bonding brackets to teeth with enamel defects.
Salama et al. [52]	SBS evaluation of orthodontic brackets bonded to demineralized enamel that was precoated with resin-modified glass ionomer or resin-infiltrated.	Forty-five teeth were demineralized and divided into groups: group I: resin infiltration; group II: Clinpro XT Varnish; group III: control.	Both study groups showed higher SBS values than the control group.	Preconditioning of demineralized enamel improves bond strength.
Triwardhani et al. [62]	To analyze the influence of fluoride varnish, CPP-ACPF varnish and resin infiltration in the treatment of white spots in orthodontic patients on SBS and enamel morphology.	Sixty bovine incisors were divided into five groups: group 1: control; the rest were demineralized and then: group 2: no treatment; group 3: fluoride varnish; group 4: CPP-ACPF; group 5: infiltration.	Fluoride varnish and CPP-ACPF caused a decrease in SBS. The highest ARI value was obtained after resin infiltration.	It is recommended to perform resin infiltration before bonding brackets to teeth with white spots and to repeat it after debonding.
Veli et al. [53]	Comparison of the influence of different methods of treating enamel defects on SBS and fracture mode of orthodontic brackets.	The teeth (except the control group) were demineralized. Group II was left untreated, while the others received: GC Tooth Mousse, Bifluorid 12, microabrasion, Opalustre or ICON.	The highest SBS values were shown by the control and ICON groups.	All tested methods improve bonding to demineralized enamel. ICON application shows SBS similar to sound enamel.
Vianna et al. [61]	SBS evaluation of brackets bonded to demineralized enamel pretreated with low-viscosity ICON Infiltrant resin and glass ionomer cement, with and without aging.	Group 1: control, healthy enamel. Groups 2 and 3 demineralization + ICON; groups 4 and 5 demineralization + Clinpro XT. Groups 3 and 5 artificially aged.	All study groups had similar or higher SBS than the control group. The highest value was shown in the Clinpro XT group without aging.	Neither ICON nor Clinpro XT treatment negatively affects SBS.
Yetkiner et al. [64]	To investigate the effect of ICON applied after hydrochloric or phosphoric acid on the adhesion of metal brackets to enamel.	For bovine enamel samples the following protocols were used: 1) H3PO4 + Transbond XT; 2) H3PO4 + Ikona + Transbond XT; 3) HCl + ICON + Transbond XT 4) HCl + ICON + Heliobond + Transbond XT.	No significant difference in SBS was observed.	ICON can be safely used with conventional adhesives on both HCl and H3PO4 etched surfaces.

**Table 2:** Detailed characteristics of studies.

Author	Type of Adhesive Used	Type of Orthodontic Bracket	Sheer Bond Strength (SBS) and Direction of the Force Used	Type of Teeth	SEM Analysis	Adhesive Remnant Index (ARI) Ratio (% of 1/2/3/4)
Veli et al. [53]	Transbond XT	Metal brackets	Vertical force SBS = 19.1 MPa	Human teeth	No data	0.2/0.35/0.4/0.05
Ekizer et al. [54]	Transbond XT	No data	Vertical force SBS = 20.6 MPa	Human teeth	No data	0.1/0.25/0.5/0.15
Montasser and Taha [55]	Transbond XT	Metal brackets	Vertical force SBS = 20.2 MPa SBS with self-etching primer (SEP) = 17.6 MPa	Human teeth	No data	0/0.1/0.1/0.8 for SEP 0/0.3/0.2/0.5
Costenoble et al. [56]	Transbond XT	No data	Vertical force SBS = 20.4 MPa	Human teeth	A homogeneously penetrating infiltration layer covering the enamel surface, copolymerizing well with the adhesive	0.75/0.08/0.08/0.08
Nimbalkar et al. [57]	Transbond XT	Metal brackets	Vertical force SBS = 16 MPa	Human teeth	No data	No data
Hammad et al. [58]	Transbond XT	Metal brackets	Vertical force SBS for Coca-Cola users = 10.39 MPa SBS for Sprite users = 11.6 MPa	Human teeth	Enamel is smoother and less eroded.	No data
Gulec et al. [46]	Transbond XT	Metal brackets	No data about force direction SBS = 4.36 MPa	Human teeth	No data	0.15/0.65/0.15/0.05
Insee et al. [47]	Transbond XT (XT); Scotchbond Universal Plus (SU); Assure PLUS (AP); Transbond Plus Self Etching (TS)	Metal brackets	Vertical force SBS XT = 11.23 MPa SBS SU = 9.52 MPa SBS AP = 8.97 MPa SBS TS = 9.14 MPa	Human teeth	No data	For XT 0/0.17/0.58/0.25 For SU 0/0.08/0.75/0.17 For AP 0/0/0.42/0.58 For TS 0/0/0.92/0.08
Baka et al. [48]	Transbond XT	Metal brackets	Vertical force SBS = 10.06 MPa	Human teeth	Performed, no description	0.5/0.3/0.15/0.05
Anicic et al. [49]	Transbond XT (XT); Scotchbond Universal (SU); Assure PLUS (AP)	Metal brackets	Vertical force SBS XT = 12.99 MPa SBS SU = 10.23 MPa SBS AP = 20.28	Human teeth	No data	for XT 0.25/0.58/0.17/0 For SU 0.33/0.58/0.08/0 for AP 0.33/0.33/0.25/0.08
Al-Mayali et al. [51]	Light cure composite adhesive	Metal brackets	No data about force direction SBS = 11.34 MPa	Human teeth	No data	No data
Salama et al. [52]	Transbond XT	Metal brackets	Vertical force SBS = 22.91 MPa	Human teeth	The damage in the adhesive-to-enamel connection was prevalent.	No data
Al-Mayali [50]	Ortho Technology adhesive (OT) 3M adhesive (3M)	Metal brackets	Vertical force SBS OT = 14.76 MPa SBS 3M = 22.54 MPa	Human teeth	No data	for OT 0.42/0.25/0.33/0 Fo 3M 0.67/0.17/0.08/0.08
Mews et al. [63]	Transbond XT	Metal brackets	Vertical force SBS = 18.6 MPa	Bovine teeth	No data	0/0.03/0.55/0.43
Triwardhani et al. [62]	Resin-modified glass ionomer	Metal brackets	No data about force direction SBS = 9.33 MPa	Bovine teeth	Irregular porosity and interconnected pores or damaged honeycomb. Less than in the control group.	No precise data
Attin et al. [60]	Transbond XT	Metal brackets	Vertical force SBS = 17 MPa	Bovine teeth	No data	No data
Vianna et al. [61]	Transbond XT	Metal brackets	Vertical force SBS = 10.61 MPa	Bovine teeth	No data	No data
Naidu et al. [59]	Heliosit (HO); Transbond XT (XT); Transbond Plus Self Etching (TS); Fuji Ortho (FO); Concise (CO)	Metal brackets	Vertical force no numerical value of SBS provided	Bovine teeth	No data	for HO 0.73/0.2/0.07/0 For XT 0.67/0.27/0.07/0 for TS 0/0.5/0.5/0 For FO 0.8/0.13/0.7/0 For CO 0.73/0.13/0.13/0
Yetkiner et al. [64]	Transbond XT	Metal brackets	No data about force direction SBS = 42.4 MPa	Bovine teeth	No enamel damage was observed.	0.95/0.05/0/0

ARI—Adhesive Remnant Index, SBS—Shear Bond Strenght, SEM—Scanning Electron Microscope, MPa—Mega Pascales, SEP—Self-etching primer, XT—Transbond XT, SU—Scotchbond Universal, AP—Assure Plus, TS—Transbond Plus Self Etching, OT—Ortho Techonology Adhesive, 3M—3M Adhesive, HO—Heliosit, FO—Fuji Ortho, CO—Concise.

**Table 3:** Quality assessment—JBI checklist for quasi-experimental studies (nonrandomized experimental studies) [42].

Authors	Is It Clear in the Study What Is the ‘Cause’ and What Is the ‘Effect’?	Were the Participants Included in Any Comparisons Similar?	Were the Participants Included in Any Comparisons Receiving Similar Treatment/Care, Other than the Exposure or Intervention of Interest?	Was There a Control Group?	Were There Multiple Measurements of the Outcome Both Pre and Post the Intervention/Exposure?	Was Follow up Complete and If Not, Were Differences Between Groups in Terms of Their Follow up Adequately Described and Analyzed?	**Were the Outcomes of Participants Included in Any Comparisons Measured in the Same Way?**	**Were Outcomes Measured in a Reliable Way?**	**Was Appropriate Statistical Analysis Used?**
Al-Mayali [50]	Yes	Yes	No	Yes	Yes	Yes	Yes	Yes	Yes
Al-Mayali et al. [51]	Yes	Yes	No	Yes	Yes	Yes	Yes	Yes	Yes
Anicic et al. [49]	Yes	Yes	No	Yes	Yes	Yes	Yes	Yes	Yes
Attin et al. [60]	Yes	Yes	No	Yes	Yes	Yes	Yes	Yes	Yes
Baka et al. [48]	Yes	Yes	No	Yes	Yes	Yes	Yes	Yes	Yes
Costenoble et al. [56]	Yes	Yes	No	Yes	Yes	Yes	Yes	Yes	Yes
Ekizer et al. [54]	Yes	Yes	No	Yes	Yes	Yes	Yes	Yes	Yes
Gulec et al. [46]	Yes	Yes	No	Yes	Yes	Yes	Yes	Yes	Yes
Hammad et al. [58]	Yes	Yes	No	No	Yes	Yes	Yes	Yes	Yes
Insee et al. [47]	Yes	Yes	No	Yes	Yes	Yes	Yes	Yes	Yes
Mews et al. [63]	Yes	Yes	No	Yes	Yes	Yes	Yes	Yes	No
Montasser and Taha [55]	Yes	Yes	No	Yes	Yes	Yes	Yes	Yes	Yes
Naidu et al. [59]	Yes	Yes	No	No	Yes	Yes	Yes	Yes	No
Nimbalkar et al. [57]	Yes	Yes	No	Yes	Yes	Yes	Yes	Yes	Yes
Salama et al. [52]	Yes	Yes	No	Yes	Yes	Yes	Yes	Yes	Yes
Triwardhani et al. [62]	Yes	Yes	No	Yes	Yes	Yes	Yes	Yes	No
Veli et al. [53]	Yes	Yes	No	Yes	Yes	Yes	Yes	Yes	Yes
Vianna et al. [61]	Yes	Yes	No	Yes	Yes	Yes	Yes	Yes	Yes
Yetkiner et al. [64]	Yes	Yes	No	No	Yes	Yes	Yes	Yes	Yes

**Table 4:** Numerical data with division into groups used for meta-analysis.

Author	Adhesive	Enamel	Number of Teeth	Mean SBS [Mpa]	SBS S.D	Control—Number of Teeth	Control—Mean SBS	Control—SBS S.D.
Montasser and Taha [55]	Transbond XT	Sound	10	20.2	4.6	10	21.1	7.5
Montasser and Taha [55]	Transbond XT	Sound	10	17.6	4.1	10	20.2	4.0
Hammad et al. [58]	Transbond XT	Sound	15	10.4	0.6	15	7.6	0.4
Hammad et al. [58]	Transbond XT	Sound	15	11.6	0.5	15	8.6	0.4
Yetkiner et al. [64]	Transbond XT	Sound	20	42.6	15.5	20	45.6	7.9
Al-Mayali [50]	Ortho Technology adhesive	Sound	12	14.8	4.1	12	13.0	2.1
Al-Mayali et al. [51]	Light cure composite adhesive	Sound	24	11.3	2.4	24	8.4	1.7
Al-Mayali [50]	3M adhesive	Sound	12	22.5	6.0	12	17.3	4.4
Costenoble et al. (2016)	Transbond XT	Eroded	12	20.4	5.0	12	26.2	8.6
Veli et al. [53]	Transbond XT	Demineralized	20	19.1	1.4	20	18.8	2.0
Ekizer et al. [54]	Transbond XT	Demineralized	20	20.6	4.4	20	12.3	1.3
Nimbalkar et al. [57]	Transbond XT	Demineralized	15	16.0	5.2	15	13.5	6.6
Gulec et al. [46]	Transbond XT	Demineralized	20	4.4	2.2	20	16.8	4.8
Insee et al. [47]	Transbond XT	Demineralized	12	11.2	3.1	12	11.7	3.2
Baka et al. [48]	Transbond XT	Demineralized	20	10.1	2.1	20	10.2	2.3
Anicic et al. [49]	Transbond XT	Demineralized	12	13.0	5.5	12	11.3	4.4
Salama et al. [52]	Transbond XT	Demineralized	15	22.9	4.1	15	17.9	5.3
Mews et al. [63]	Transbond XT	Demineralized	40	18.6	4.4	40	17.9	4.2
Attin et al. [60]	Transbond XT	Demineralized	12	17.0	3.4	12	30.8	5.1
Vianna et al. [61]	Transbond XT	Demineralized	15	10.6	1.8	15	11.5	2.3
Insee et al. [47]	Transbond Plus Self Etching	Demineralized	12	9.1	0.7	12	11.7	3.2
Insee et al. [47]	Scotchbond Universal Plus	Demineralized	12	9.5	1.7	12	11.7	3.2
Anicic et al. [49]	Scotchbond Universal	Demineralized	12	10.2	2.8	12	11.3	4.4
Triwardhani et al. [62]	Resin-modified glass ionomer	Demineralized	12	9.3	1.0	12	8.7	1.1
Insee et al. [47]	Assure PLUS	Demineralized	12	9.0	1.1	12	11.7	3.2
Anicic et al. [49]	Assure PLUS	Demineralized	12	20.3	3.4	12	11.3	4.4

## Data Availability

Data sharing is not applicable to this article as no new data were created or analyzed in this study.
